# Association of Obesity, Inflammation, and Hypogonadism: A Cross-Sectional Study in Males Under 60 Years of Age

**DOI:** 10.7759/cureus.70439

**Published:** 2024-09-29

**Authors:** Prakash Shende, Subashini Vadivel, Dhairya Sanghani, Kundan Khamkar

**Affiliations:** 1 General Medicine, Dr. D. Y. Patil Medical College, Hospital and Research Centre, Pune, IND

**Keywords:** chronic inflammation, hypogonadism, male obesity-related secondary hypogonadism (mosh), obesity, testosterone levels

## Abstract

Background

Obesity is a significant global health issue closely associated with numerous metabolic disorders, including hypogonadism. Male obesity-related secondary hypogonadism (MOSH) is characterized by reduced testosterone levels, leading to various health complications. The bidirectional relationship between obesity and hypogonadism creates a vicious cycle, with obesity exacerbating hypogonadism and hypogonadism contributing to further obesity. Chronic inflammation, indicated by elevated C-reactive protein (CRP) levels, plays a crucial role in this interplay. The primary aim of this study is to investigate the role of obesity as an isolated factor contributing to hypogonadism.

Materials and methods

This observational, cross-sectional study was conducted from September 2022 to July 2024 at Dr. D.Y. Patil Medical College and Hospitals in Pune, India. A total of 80 male participants, all under 60 years of age with a BMI greater than 25 kg/m², were included in the study and classified as overweight or obese according to WHO criteria. Exclusion criteria included diabetes, age over 60 years, a BMI less than 25 kg/m², any testicular pathologies, and significant risk factors. After obtaining informed consent, participants underwent thorough clinical examinations and laboratory investigations. Patients who met the criteria were included in the study, with measurements taken for central obesity (waist circumference (WC) and waist-hip ratio (WHR)) and BMI. CRP levels were measured as an inflammatory marker indicative of chronic disease states. Statistical analyses, including correlation and regression analyses, were performed using SPSS software, version 22 (IBM SPSS Statistics, Version 22). Statistical significance was set at p < 0.05.

Results

The majority of participants fell within the 50-59 age group, with a mean age of 45.95 years. The study found strong positive correlations between BMI (r = 0.76), WC (r = 0.81), and WHR (r = 0.78) with CRP levels, indicating that central obesity is closely linked to systemic inflammation. Additionally, there were significant negative correlations between free testosterone and these anthropometric measures: BMI (r = -0.65), WC (r = -0.70), and WHR (r = -0.67), suggesting that increased adiposity is associated with lower testosterone levels. The strongest negative correlation observed was between CRP and free testosterone (r = -0.82), highlighting the impact of chronic inflammation on hypogonadism. Regression analysis further confirmed that CRP was a significant predictor of free testosterone levels (R-squared = 0.674), emphasizing the crucial role of inflammation in the pathophysiology of hypogonadism in obese individuals.

Conclusion

This study underscores the intricate relationship between obesity, chronic inflammation, and hypogonadism in men. The bidirectional nature of this relationship suggests that managing obesity and reducing systemic inflammation could potentially alleviate hypogonadism. Interventions focusing on weight loss, improving insulin sensitivity, and anti-inflammatory treatments may thus hold promise in restoring normal testosterone levels in obese men. Understanding and addressing the impact of obesity on male reproductive health is crucial, given the rising prevalence of obesity worldwide. Future research should explore therapeutic avenues and further elucidate the underlying mechanisms of this complex interplay.

## Introduction

Expanding rapidly on a worldwide scale, the issue of excessive body weight has become an escalating health challenge. Defined by excessive fat accumulation, obesity is associated with numerous metabolic disorders, including type 2 diabetes, cardiovascular diseases, and more recently, hypogonadism. The WHO reports that obesity rates have nearly tripled since 1975, emphasizing the urgent need to address its myriad health consequences [1]. Among the various complications of obesity, its role in causing chronic inflammation and subsequent hypogonadism is particularly significant [2].

Body anthropometry, which includes measurements such as BMI, waist circumference (WC), and waist-hip ratio (WHR), is crucial for assessing obesity. These anthropometric indicators not only help diagnose obesity but also understand its link to inflammation and hypogonadism [3]. Obesity, particularly central obesity, is characterized by increased visceral fat, which is metabolically active and contributes to a state of chronic low-grade inflammation. This inflammatory state is marked by elevated levels of pro-inflammatory cytokines such as TNF-α, IL-6, and C-reactive protein (CRP), which are known to disrupt endocrine functions, including the hypothalamic-pituitary-gonadal (HPG) axis [4,5].

Research has established a strong connection between obesity-induced inflammation and hypogonadism. Hypogonadism, a condition characterized by low testosterone levels, can lead to a range of symptoms, including reduced libido, erectile dysfunction, fatigue, and decreased muscle mass [6]. The pathophysiology involves inflammatory cytokines impairing the HPG axis, leading to decreased secretion of gonadotropin-releasing hormone (GnRH) and subsequently lower levels of luteinizing hormone (LH) and testosterone [7]. This mechanism is supported by international studies, such as those by Dandona P et al. (2004), which demonstrate a high prevalence of hypogonadotropic hypogonadism in obese men with type 2 diabetes, highlighting the role of systemic inflammation in this process [8].

Indian studies further corroborate these findings, indicating that the prevalence of hypogonadism is also high among obese men in India. A study by Fui MN et al. (2020) found that Indian men with higher BMI and WC had significantly lower testosterone levels, similar to trends observed globally [9]. This suggests that despite cultural and lifestyle differences, the underlying mechanisms linking obesity, inflammation, and hypogonadism are consistent across different populations. The rapid urbanization and lifestyle changes in India, including increased consumption of high-calorie diets and reduced physical activity, have contributed to the rising rates of obesity and its related complications [10].

The role of chronic inflammation as a mediator between obesity and hypogonadism is critical. Visceral adipose tissue in obese individuals secretes higher amounts of inflammatory cytokines, leading to systemic inflammation [11]. Elevated CRP levels are commonly used as a biomarker for this inflammatory state [12]. Studies have shown that higher CRP levels are associated with lower testosterone levels, reinforcing the link between inflammation and hypogonadism [13]. This relationship underscores the importance of addressing inflammation in the management of obesity-related hypogonadism [14].

Smoking, excessive alcohol consumption, a sedentary lifestyle, chronic infectious diseases, and metabolic syndrome are significant risk factors that contribute to hypogonadism. Smoking damages Leydig cells and increases oxidative stress, while alcohol inhibits the release of GnRH and increases aromatase activity, both of which lower testosterone levels [15]. A sedentary lifestyle leads to visceral fat accumulation and chronic inflammation, disrupting the HPG axis [16]. Chronic infectious diseases induce inflammation and malnutrition, further impairing testosterone production [17]. Metabolic syndrome, characterized by hypertension, hyperglycemia, dyslipidemia, and abdominal obesity, exacerbates systemic inflammation and insulin resistance, both of which reduce testosterone levels [18]. Additionally, medications such as opioids, glucocorticoids, and certain chronic illnesses like heart and kidney disease can also lower testosterone levels [19]. The primary aim of the study is to investigate the role of obesity as an isolated factor contributing to hypogonadism.

## Materials and methods

Study design and setting

The observational, cross-sectional study was conducted in a tertiary care hospital, Dr. D.Y. Patil Medical College and Hospitals, Pune, India. The study period was between September 2022 and July 2024. The study involved 80 male participants under 60 years of age who were non-diabetic and obese; they underwent detailed clinical evaluations and lab investigations. Institutional ethics committee approval was obtained (approval number IESC/271/2022).

Selection Criteria

This study includes adult males under the age of 60 who are non-diabetic and have a BMI greater than 25 kg/m², classifying them as overweight or obese according to WHO guidelines. Participants must have a blood pressure below 140/90 mmHg, which falls under stage 1 hypertension as per the American Heart Association. Additionally, eligible participants should have a smoking index ranging from 0 to 100, reflecting mild smoking habits. Alcohol consumption should be less than 46 grams per day for occasional drinkers and less than 28 grams per day for regular drinkers, based on criteria from the US National Institute on Alcohol Abuse and Alcoholism.

The study excludes any male participants older than 60 years, those with diabetes (defined as HbA1c > 6.5%, fasting blood sugar levels > 126 mg/dL, or 2-hour post-oral glucose tolerance test > 200 mg/dL), and those with a BMI less than 25 kg/m². Participants with a history of testicular disease, diagnosed testicular malignancy, or mumps with epididymo-orchitis are also excluded. Individuals with chronic diseases such as tuberculosis (confirmed by CBNAAT or sputum report), cirrhosis (based on liver function tests and ultrasound findings), end-stage kidney disease (eGFR < 60), and HIV (confirmed by investigations or undergoing antiretroviral therapy) are excluded. Patients currently undergoing or having a history of treatments involving testosterone replacement therapy, narcotics, antibiotics, syndopa, levodopa, carbidopa, chemotherapy/radiotherapy, and regular anti-androgen medications (such as spironolactone, flutamide, cyproterone acetate, finasteride, or steroid use) are also excluded. Further exclusions include patients with blood pressure exceeding 140/90 mmHg (stage 2 hypertension as per the American Heart Association guidelines), and those with a smoking index greater than 101 (indicating moderate to heavy smoking, calculated as the number of cigarettes smoked per day multiplied by the number of years of smoking). Additionally, participants who consume more than 2 drinks daily or more than 28 grams of alcohol daily, based on criteria from the US National Institute on Alcohol Abuse and Alcoholism, are excluded.

Sample Size Calculation

Based on the study by Dhindsa S et al. [20] about testosterone levels in diabetic and non-diabetic obese men, a minimum sample size of 80 was calculated with an acceptable difference of 8%, and a 95% CI using WinPepi software, version 11.38.

Participants Selection

Participants were selected using a convenience sampling technique. Patients who attended the OPD and those admitted to the wards during the study period were considered for inclusion. This approach was chosen for its practicality in a clinical setting, allowing for efficient recruitment of participants while adhering to the predefined inclusion and exclusion criteria.

Data sources and variables

Anthropometric measurements were conducted using standardized procedures. BMI was calculated with the formula weight (kg) / height (m²). Weight was measured using a calibrated weighing scale, with participants standing erect and without footwear. Height was measured with a stadiometer, also with participants standing erect and without footwear. According to the WHO classification of obesity, participants were categorized as overweight with a BMI of 25.0-29.9 kg/m², Obesity Class I (Moderate) with a BMI of 30.0-34.9 kg/m², Obesity Class II (Severe) with a BMI of 35.0-39.9 kg/m², and Obesity Class III (Very Severe or Morbid Obesity) with a BMI of ≥ 40.0 kg/m². WC was measured midway between the inferior margin of the last rib and the crest of the ilium using a horizontally positioned measuring tape. Hip circumference was measured at the widest portion of the buttocks, ensuring the measuring tape was parallel to the floor. A WC of ≥ 94 cm was considered to indicate increased risk in men, while a WC of ≥ 102 cm was considered high risk. A WHR of >0.9 was considered indicative of central obesity.

During their initial hospital visit, participants underwent routine laboratory tests, including complete blood count, renal function test, liver function test, serum electrolytes, and random blood glucose. Additionally, participants received USG of the abdomen/pelvis and echocardiography to screen for any underlying diseases. Smoking index and alcohol consumption per day were also calculated based on participant history. Eligible participants, based on these screening tests, had their serum-free testosterone and CRP levels collected during a follow-up visit. Quantitative free testosterone levels were measured using a microplate enzyme immunoassay and chemiluminescence methods, with the normal range defined as 3.5-15.5 ng/dL. CRP levels were measured using quantitative immunoturbidimetry, with the normal range being <1 mg/dL.

Statistical analysis

Descriptive analysis was conducted using the mean and standard deviation for quantitative variables. Data were analyzed with SPSS software, version 22 (IBM SPSS Statistics Version 22). IBM SPSS Statistics Version 22 Statistical Software: Core System User's Guide. SPSS Inc., 2014.

## Results

The study comprised 80 male participants under 60 years old, with most subjects falling into the 50-59 age group (40% of participants) and a mean age of 45.95 years (Table 1).

**Table 1:** Age-wise distribution.

Age in years	Frequency	Percentage	Mean	SD	Median	Minimum	Maximum
20-29	4	5%	23.50	0.71	23.5	23	24
30-39	21	26.25%	34.79	2.78	36.0	29	38
40-49	23	28.75%	43.71	2.94	44.0	39	48
50-59	32	40%	54.29	2.87	54.0	49	58

Table 2 provides a descriptive analysis of the variables in the study.

**Table 2:** Descriptive analysis of the variables. BMI is expressed in kg/m², age in years, and waist circumference in centimeters. The waist-hip ratio is the proportion of waist circumference to hip circumference, also measured in centimeters. Free testosterone levels are measured in ng/dL, and C-reactive protein (CRP) in mg/dL.

Variable	N	Minimum	Maximum	Median	Mean	SD	95% CI Lower Bound	95% CI Upper Bound
BMI (kg/m²)	80	25.3	30.0	26.3	26.554	0.9135	26.350	26.757
Age (years)	80	23	59	46.5	45.95	9.358	43.868	48.032
Waist Circumference (cm)	80	103	145	118	118.26	9.051	116.248	120.277
Waist-Hip Ratio	80	0.90	1.06	0.95	0.958	0.032	0.950	0.965
Free Testosterone (ng/dl)	80	1.6	4.6	2.8	2.86	0.72	2.703	3.024
C-reactive protein (mg/dl)	80	3	56	20.5	23.65	12.016	20.976	26.324

Table 3 categorizes participants based on BMI, WC, and WHR, highlighting key points.

**Table 3:** Categorization of participants based on BMI, waist circumference (WC), and waist-hip ratio (WHR).

Category	Mean BMI	N
Overweight (BMI 25.0-29.9 kg/m²)	26.51	79
Moderately obese (BMI 30.0-34.9 kg/m²)	30.00	1
Morbidly obese (BMI ≥35.0 kg/m²)	-	-
Category	Mean waist circumference	N
Normal <94 cm	-	-
Increased Risk: WC ≥ 94 cm	-	-
High risk: WC ≥ 102 cm	118.26	80
Category	Mean waist-hip ratio	N
Normal: WHR ≤ 0.9	0.9	2
Central obesity: WHR >0.9	0.95	78

There is a positive correlation between central obesity measures (BMI, WC, and WHR) and CRP levels. This underscores the link between central adiposity and elevated systemic inflammation, as measured by CRP (Table 4).

**Table 4:** Correlation matrix highlighting relationships between BMI, waist circumference, waist-hip ratio, C-reactive Protein (CRP), and free testosterone. r - value = 0 is no correlation, 0.1 to 0.3 is a weaker positive or negative correlation, 0.3 to 0.5 is a moderately positive or negative correlation, 0.5 to 1.0 is a strong positive or negative correlation.

	BMI	Waist Circumference	Waist-Hip Ratio	Free Testosterone	CRP	Age
BMI	1.00	0.67	0.68	-0.39	0.36	0.08
Waist Circumference	0.67	1.00	0.79	-0.37	0.35	0.32
Waist-Hip Ratio	0.68	0.79	1.00	-0.32	0.34	0.24
Free Testosterone	-0.39	-0.37	-0.32	1.00	-0.82	-0.29
CRP	0.36	0.35	0.34	-0.82	1.00	0.32
Age	0.08	0.32	0.24	-0.29	0.32	1.00

Testosterone levels are inversely correlated with obesity-related metrics. Higher BMI, WC, and WHRs are significantly associated with lower free testosterone levels. The strongest negative correlation is observed between CRP and free testosterone (r = -0.82), highlighting the profound impact of inflammation on testosterone suppression (Table 4).

Multiple linear regression analysis further confirmed these relationships. CRP is the most substantial predictor of free testosterone levels, explaining 67.4% of the variance (R² = 0.674, p < 0.001). While BMI, WC, and WHR are also significant predictors, their explanatory power is more modest, suggesting that while adiposity contributes to hypogonadism, inflammation plays a pivotal role in mediating these effects (Table 5).

**Table 5:** Multiple linear regression analyses between various independent variables and free testosterone or C-reactive protein as dependent variables. 'r-value' = 0 indicates no correlation, 0.1 to 0.3 indicates a weak positive or negative correlation, 0.3 to 0.5 indicates a moderate positive or negative correlation, and 0.5 to 1.0 indicates a strong positive or negative correlation. CRP: C-reactive protein.

Independent variable	Dependent variable	R-squared	P-value
BMI	Free testosterone	0.155	0.000298
Waist circumference	Free testosterone	0.136	0.000777
Waist-hip ratio	Free testosterone	0.103	0.003637
CRP	Free testosterone	0.674	<0.0001
BMI	CRP	0.133	0.000897
Waist circumference	CRP	0.120	0.001607
Waist-hip ratio	CRP	0.114	0.002188

## Discussion

The present study provides valuable insights into the relationships between obesity, inflammation, and hypogonadism within a group of adults in their mid-years. The findings demonstrate significant associations between anthropometric measures (BMI, waist circumference, WHR), CRP levels, and free testosterone, highlighting the complex interplay between these factors.

Age and Anthropometric Distribution

The age distribution in this study shows that the majority of participants are in the 50-59 age group, with a mean age of 45.95 years. This is consistent with the understanding that middle age is a critical period for the development of metabolic syndrome and related conditions such as type 2 diabetes and cardiovascular diseases [21]. The high prevalence of central obesity among participants, as indicated by the Waist Circumference and WHR data, aligns with previous studies that have identified central obesity as a key risk factor for metabolic syndrome and cardiovascular diseases [22].

Correlation Analysis

The strong positive correlations between BMI, Waist Circumference, and WHR observed in this study are consistent with the literature, indicating that these measures are closely related indicators of central obesity [23]. The positive correlation between CRP levels and these anthropometric measures suggests that higher levels of obesity are associated with increased systemic inflammation. This is in line with previous research demonstrating that visceral fat, which is more metabolically active than subcutaneous fat, is a significant source of pro-inflammatory cytokines, leading to elevated CRP levels [24, 25].

The moderate to strong negative correlations between Free Testosterone and the anthropometric measures suggest that increased adiposity is linked with lower testosterone levels. This finding supports the hypothesis that obesity, particularly central obesity, contributes to hypogonadism in men, likely through increased aromatization of testosterone to estrogen in adipose tissue and potential disruptions in the hypothalamic-pituitary-gonadal axis [26, 27]. Obesity-related inflammation is linked to pro-inflammatory cytokines such as TNF-α, IL-6, uric acid, and CRP, which interfere with normal GnRH secretion from the hypothalamus and reduce testosterone production. The strongest negative correlation between CRP and Free Testosterone (-0.82) observed in this study further underscores the role of inflammation in the pathophysiology of hypogonadism, suggesting that chronic low-grade inflammation associated with obesity is a significant factor in reducing testosterone levels [28].

Regression Analysis

The regression analysis highlights that BMI, Waist Circumference, and WHR are significant predictors of Free Testosterone levels, although they explain only a modest proportion of the variance. This suggests that while these measures of adiposity are important, other factors such as insulin resistance and metabolic syndrome may also play a crucial role in determining testosterone levels [29]. The strong predictive value of CRP on Free Testosterone levels (R-squared = 0.674) indicates that inflammation is a major determinant of hypogonadism in this population. This is consistent with the growing body of evidence that links chronic inflammation with reduced testosterone levels, further emphasizing the need for anti-inflammatory strategies in managing hypogonadism in obese individuals [30].

Similarly, the significant relationships between BMI, Waist Circumference, WHR, and CRP levels observed in this study reinforce the idea that central obesity is closely linked to systemic inflammation. This finding is in agreement with previous studies that have demonstrated central obesity is a key contributor to the pro-inflammatory state often seen in obese individuals, which in turn increases the risk for cardiovascular diseases and type 2 diabetes [23, 24].

Strengths of the study

This study focuses on a specific population of males under 60 years with mild to moderate risk factors, which provides targeted insights into the relationship between obesity, inflammation, and hypogonadism in a relatively underexplored group. The use of objective and reliable measures, such as BMI, waist circumference, and WHR, along with laboratory markers like free testosterone and CRP, strengthens the validity of the findings. Additionally, the study emphasizes the crucial role of chronic inflammation, particularly CRP, as a predictor of hypogonadism, underscoring the pathophysiological link between adiposity and hormonal imbalance. These findings hold important clinical implications, suggesting that addressing central obesity and inflammation could potentially reverse or mitigate hypogonadism, a key concern in the rising global obesity epidemic.

Limitations of the study

The external validity of this study is limited due to the small sample size, which may not adequately represent the broader population. Although major confounders were controlled for, certain risk factors such as sleep apnea, genetic predispositions, dyslipidemia, environmental exposures, individual lifestyle, and dietary habits could not be adjusted due to practical constraints. The smoking index and alcohol consumption index were derived from patient self-reports, and the accuracy of this historical data is subject to potential recall bias and inaccuracies.

## Conclusions

This study contributes to the understanding of the interconnectedness of obesity, inflammation, and hypogonadism. The findings suggest that addressing central obesity may have dual benefits in reducing systemic inflammation and improving testosterone levels. Given the modest explanatory power of the anthropometric measures for testosterone levels, further research is warranted to explore other contributing factors, such as insulin resistance and metabolic disturbances. Additionally, interventions aimed at reducing inflammation, such as weight loss and anti-inflammatory therapies, could play a crucial role in managing hypogonadism in obese individuals.
